# α2-Antiplasmin as a potential regulator of the spatial memory process and age-related cognitive decline

**DOI:** 10.1186/s13041-020-00677-3

**Published:** 2020-10-15

**Authors:** Eri Kawashita, Keiichi Ishihara, Haruko Miyaji, Yu Tanishima, Akiko Kiriyama, Osamu Matsuo, Satoshi Akiba

**Affiliations:** 1 Department of Pathological Biochemistry, Kyoto Pharmaceutical University, 5, Nakauchi-cho Misasagi, Yamashina-ku, Kyoto, 607-8414 Japan; 2 Department of Pharmacokinetics, Faculty of Pharmaceutical Science, Doshisha Women’s College of Liberal Arts, Kodo, Kyotanabe-shi, Kyoto, 610-0395 Japan; 3 Faculty of Medicine, Kindai University, 377-2 Ohnohigashi, Osakasayama, 589-8511 Japan

**Keywords:** α2-Antiplasmin, Brain aging, Hippocampus, Neurogenesis, Plasmin, Spatial memory

## Abstract

α2-Antiplasmin (α2AP), a principal physiological plasmin inhibitor, is mainly produced by the liver and kidneys, but it is also expressed in several parts of the brain, including the hippocampus and cerebral cortex. Our previous study demonstrated that α2AP knockout mice exhibit spatial memory impairment in comparison to wild-type mice, suggesting that α2AP is necessary for the fetal and/or neonatal development of the neural network for spatial memory. However, it is still unclear whether α2AP plays a role in the memory process. The present study demonstrated that adult hippocampal neurogenesis and remote spatial memory were enhanced by the injection of an anti-α2AP neutralizing antibody in WT mice, while the injection of α2AP reduced hippocampal neurogenesis and impaired remote spatial memory, suggesting that α2AP is a negative regulator in memory processing. The present study also found that the levels of α2AP in the brains of old mice were higher than those in young mice, and a negative correlation between the α2AP level and spatial working memory. In addition, aging-dependent brain oxidative stress and hippocampal inflammation were attenuated by α2AP deficiency. Thus, an age-related increase in α2AP might cause cognitive decline accompanied by brain oxidative stress and neuroinflammation. Taken together, our findings suggest that α2AP is a key regulator of the spatial memory process, and that it may represent a promising target to effectively regulate healthy brain aging.

## Introduction

α2-Antiplasmin (α2AP) is a member of the serine protease inhibitor (serpin) family, and a principal physiological plasmin inhibitor [1]. Lysine residues at the C-terminus of α2AP bind to lysine-binding sites in the kringle domains of plasmin and its precursor, plasminogen to form plasmin-antiplasmin complexes [2, 3]. Thus, α2AP regulates fibrinolysis and proteolysis. α2AP circulates at a concentration of approximately 70 μg/mL (1 μM), and mainly produced by the liver and kidneys; but it is also expressed in several parts of the brain, including the hippocampus and cortex [4, 5].

In addition to α2AP, plasmin presents in the mouse brain [6, 7], and degrades extracellular matrix components, including laminin and proteoglycan [8–11]. It also cleaves neurotrophins, such as brain-derived neurotrophic factor (BDNF) and nerve growth factor (NGF), and converts the precursor form into the mature form [12, 13]. Plasmin has been suggested to play both protective and toxic roles in the brain. The proteolysis of extracellular matrix by plasmin leads to the enhancement of dendritic spine motility and neuritogenesis [14, 15]; and the conversion by plasmin from the precursor form to the mature form of BDNF is essential for long-term hippocampal plasticity [12]. On the other hand, the degradation of laminin by plasmin results in the impairment of a late phase of long-term potentiation (LTP) in the hippocampus [10]; excess tissue plasminogen activator (tPA)/plasmin suppresses dendritogenesis and synaptogenesis in Purkinje neurons [16]. Moreover, plasmin disrupts mossy fiber axon guidance [17]. In addition, the tPA/plasmin proteolytic cascade promotes neuronal cell death in the hippocampus [9, 11]. Therefore, α2AP might be an important regulator of these actions of plasmin; however, the physiological and pathological roles of α2AP in the brain have not been sufficiently investigated.

Our previous study demonstrated that the length of dendrites was markedly shorter and the number of dendritic branches was markedly lower in the hippocampal neurons from α2AP knockout (α2AP^−/−^) mice in comparison to α2AP^+/+^ (wild-type; WT) mice [18]. Given that excessive plasmin activity suppresses dendritogenesis in Purkinje neurons [16], the control of plasmin activity by α2AP is critical to dendritic growth. We also found that exogenous treatment with α2AP can somewhat enhance dendritic elongation and branching in the hippocampal neurons from WT mice, without the inhibition of plasmin [18]. Thus, α2AP is considered to regulate the dendritic growth in the neurons both in plasmin-dependent and plasmin-independent manners. Furthermore, we demonstrated that the α2AP^−/−^mice exhibit impaired memory, including working memory, spatial memory and fear conditioning memory, in comparison to WT mice [19]. Considering these findings, α2AP is likely to be necessary for the fetal and neonatal development of the neural network for memory functions; however, it is still unclear whether α2AP plays a role in memory process.

It is widely accepted that the hippocampus is a crucial brain region for learning and memory. Adult neurogenesis in the hippocampus is one of the most important mechanisms for the spatial memory process; the inhibition of adult neurogenesis by irradiation impairs long-term spatial memory, while the enhancement of neurogenesis after running facilitates LTP and spatial memory [20, 21]. Furthermore, adult hippocampal neurogenesis contributes to contextual fear memory [22]. It is also known that the hippocampal structure and function are especially vulnerable to aging. Age-related decreases in neurogenesis and synaptic plasticity accompanied by oxidative stress and neuroinflammation are suggested to lead to cognitive decline [23]. α2AP is expressed at a higher level in the hippocampus in comparison to other parts of the brain [4]. Based on the distribution, α2AP might play an important role in the hippocampus-dependent spatial memory process and age-related cognitive decline. The present study provides evidence that α2AP is a negative regulator of adult hippocampal neurogenesis and spatial memory, and furthermore, that α2AP is associated with brain aging.

## Materials and methods

### Animals

Male 12-week-old C57BL/6J mice were purchased from Japan Charles River (Yokohama, Japan). α2AP-deficient (α2AP^−/−^) mice were generated by homologous recombination using embryonic stem cells, as described previously [24, 25], and were repeatedly backcrossed to C57BL/6J mice for more than 10 generations. All experiments were approved by the institutional animal care and use committee of Kyoto Pharmaceutical University (permit number: 18-16-017) and were performed in accordance with the institutional guidelines. All efforts were made to minimize suffering.

### Y-maze test

The Y-maze apparatus consisted of three arms, the walls of which had different markings. The mice were placed into the center and allowed to explore the apparatus for 8 min, while being monitored by a video-tracking system (Ethovision XT; Noldus Company, Wageningen, The Netherlands). The alteration of their behavior was calculated as the ratio of the number of alterations to the total number of arm entries minus 2.

### Morris water maze (MWM) test

Mice received visible platform pre-training on the first day, followed by hidden platform training for two days. In the hidden platform training, two sessions consisting of four trials per session were performed on two days. Mice were placed into the pool from four different directions in each of the four trials, and the escape latency was measured by a video-tracking system (SMART; Panlab, Spain). The second day of training was performed the following day, followed by a 60-s probe test without a platform. The time in each quadrant was analyzed by a video-tracking system (SMART). To evaluate long-term memory, the probe tests were performed 1 and 3 months after hidden platform training.

### Intracerebroventricular injection

After the first day of MWM training, mice were anesthetized with 1.8–1.9% isoflurane, and then were injected 200 nM of α2AP (Calbiochem, MA, USA) or 0.1 μg/μL of an anti-α2AP neutralizing goat antibody (AF1470, R&D System, MN, USA) as well as saline or normal goat IgG control, respectively (AB-108-C, R&D System) in a total volume of 20 μL into the lateral ventricle (0.5 mm caudally and 1.0 mm laterally to the bregma and 2.0 mm vertically from the skull surface) with a two-step needle (Star needle; Seiseido, Tokyo, Japan) attached to a glass syringe (As one, Osaka, Japan). After injection, the needle was held at the site for 1 min to prevent reverse flow.

### In vivo BrdU labeling

5-Bromo-2′-deoxyuridine (BrdU) (Nacalai Tesque Inc, Kyoto, Japan) was dissolved in saline, and injected intraperitoneally at 24-h intervals for 7 days at 50 mg/kg. The mice were perfused with PBS and 4% paraformaldehyde (PFA) in PBS, and the brains were then fixed in 4% PFA for 48 h, then soaked in 30% sucrose for 5 days. Thirty-micrometer-thick frozen coronal sections were prepared and stained with anti-BrdU mouse antibody (555627, BD Biosciences, Franklin Lakes, NJ, USA; diluted 1:200 with blocking solution) and anti-Ki67 rabbit antibody (ab16667, Abcam, Cambridge, UK; diluted 1:500 with blocking solution) after 2 N HCl-treatment at 37℃ for 30 min, neutralization with 0.1 M of boric acid pH 8.5 at room temperature for 10 min, and blocking with a Mouse on Mouse blocking kit (Vector Laboratories, CA, USA) and blocking solution (0.3% Triton X-100 and 10% normal goat serum (Vector Laboratories) in PBS). The sections were then incubated with anti-BrdU and anti-Ki67 antibodies at room temperature at 4 °C overnight. After washing with PBS, the sections were treated with Alexa 488-conjugated goat anti-mouse IgG (A11001, Invitrogen; diluted 1:1000 with blocking solution) and Alexa 546-conjugated goat anti-rabbit IgG (A11010, Invitrogen, CA, USA; diluted 1:1000 with blocking solution), and then coverslipped in Prolong Gold™ antifade reagent (Invitrogen). The specimens were observed using a confocal laser microscope NIKON A1R (Nikon, Tokyo, Japan).

### Immunostaining of doublecortin

Thirty-micrometer-thick frozen coronal sections were treated with 0.3% H_2_O_2_ in methanol at room temperature for 10 min. The sections in Retrievagen A (pH 6.0) (BD Biosciences) were autoclaved and washed in PBS, and then incubated with blocking solution at room temperature for 1 h, and treated with anti-doublecortin (Dcx) mouse antibody (ab18723, Abcam; diluted 1:200 with blocking solution) at 4 °C overnight. After washing with PBS, they were incubated with biotinylated anti-rabbit IgG antibody (BA-1000, Vector Laboratories; diluted 1:200 with blocking solution) at room temperature for 30 min. The detection of antibody-antigen complexes was accomplished using a Vectastain Elite ABC kit (Vector Laboratories) and Metal-Enhanced DAB Substrate kit (Thermo Scientific, IL, USA). The immunostained sections were photographed using a microscope with a digital camera (model IX71; Olympus, Tokyo, Japan). Images were taken at full resolution with a single image dimension set at 1360 × 1024 pixels.

### Immunoblotting

After perfusion with PBS, the hippocampi and cerebral cortexes from the mice were homogenized and sonicated in lysis buffer: 10 mM Tris–HCl buffer (pH 7.5) containing 1% SDS, 1% Triton X-100, and a protease inhibitor cocktail (Roche, Mannheim, Germany). The protein concentration in each lysate was measured using a BCA protein assay kit (Pierce, IL, USA). Lysates containing equal amounts of protein were subjected to SDS–polyacrylamide gel electrophoresis on a 10% acrylamide gel. Proteins were transferred onto PVDF or nitrocellulose membranes. After blocking with 3% skim milk in Tris-buffered saline containing 0.05% Tween-20 (TBS-T), the membranes were incubated with anti-α2AP goat antibody (AF1239, R&D System; diluted 1:500 with blocking solution), anti-hexanoyl-lysine (HEL) mouse antibody (MHL-021P, Japan Institute for the Control of Ageing, Shizuoka, Japan; diluted 1:500 with blocking solution), or anti-glyceraldehyde 3-phosphate dehydrogenase (GAPDH) mouse antibody (016-25523, Wako Pure Chemical Industries; diluted 1:4000 with blocking solution) at 4 °C overnight. After washing with TBS-T, the membranes were incubated with horseradish peroxidase-conjugated rabbit anti-goat IgG (P0449, Dako, Glostrup, Denmark), or goat anti-mouse IgG (A108PS, American Qualex, CA, USA; diluted 1:2500 with TBS-T or 0.3% skim milk in TBS-T) for 1 h. After washing again, immunoreactive bands were detected using Chemi-Lumi One Super (Nacalai Tesque) with an LAS-3000 mini-image analysis system (Fujifilm, Tokyo, Japan). The band intensities were quantified using the ImageJ software program. The same sample as a loading control was included in each Western blot analysis, and the band intensities were normalized.

### Enzyme-linked immunosorbent assay

The α2AP levels in the cerebrospinal fluid and plasma were measured with a mouse α2AP ELISA kit (Innovative Research, MI, USA). The cerebrospinal fluid was collected as detailed below. Briefly, a guide cannula (PEG-4; Eicom, Kyoto, Japan) was implanted into the lateral right ventricle (0.2 mm caudally and 1.1 mm laterally to the bregma; and 1.4 mm vertically from the brain surface), fixed to the skull with dental cement (Unifast III; Corp., Tokyo, Japan), and was then occluded with a dummy cannula (PED-4; Eicom). The mice were returned to their home cage and allowed to recover for 2 days. A microdialysis probe (PEP-4-01; Eicom) was inserted into the lateral right ventricle through the guide cannula under anesthesia with 1.5% isoflurane. The probe was perfused continuously at a flow rate of 10 μL/min with artificial cerebrospinal fluid (ACSF) containing 147 mM NaCl, 4 mM KCl and 3 mM CaCl_2_. The outflow fraction for the first 3 h was discarded, and then the dialysis sample perfused with ACSF at a flow rate of 1 μL/min was collected for 1 h under anesthesia with 1.5% isoflurane.

### Extraction of RNA and real-time PCR

Total RNA was isolated from the hippocampi and cerebral cortexes using TRIsure (Bioline, London, UK). After the addition of CHCl_3_, centrifugation was performed at 15,000×*g* for 15 min. The resultant supernatants were each mixed with an equal volume of 2-propanol. After centrifugation, the pellets were rinsed with 75% ethanol/diethylpyrocarbonate (DEPC)-treated water and then dried. The pellets were each dissolved in an appropriate volume of DEPC-treated water as total RNA fractions. RNA from each sample (1 µg) was transcribed using ReverTra Ace-α (Toyobo, Osaka, Japan) according to the manufacturer’s protocol. Quantitative PCR was performed to analyze the murine IL-6, TNF-α and IL-1β mRNA expression relative to the GAPDH mRNA expression using a MiniOpticon real-time PCR system (Bio-Rad Laboratories, CA, USA). We used the following primers: IL-6, 5′-GTTCTCTGGGAAATCGTGGA-3′ (sense) and 5′-GGAAATTGGGGTAGGAAGGA-3′ (antisense); TNF-α, 5′-AAATGGGCTTTCCGAATTCA-3′ (sense) and 5′-CAGGGAAGAATCTGGAAAGGT-3′ (antisense); IL-1β, 5′-CAAATCTCGCAGCAGCACA-3′ (sense) and 5′-TCATGTCCTCATCCTGGAAGG-3′ (antisense); and GAPDH, 5′-TGTGTCCGTCGTGGATCTGA-3′ (sense) and 5′-TTGCTGTTGAAGTCGCAGGAG-3′ (antisense). The fold-change in the expression levels of IL-6, TNF-α and IL-1β relative to the GAPDH expression as an endogenous control gene were determined by the − ∆Ct method.

### Statistical analysis

Data are reported as the mean ± standard error of the mean (SE). Differences among mean values were analyzed using a one-way analysis of variance (ANOVA) followed by an LSD post-hoc test or Student’s *t*-test. P values of < 0.05 were considered to indicate statistical significance.

## Results

### Enhancement of hippocampal neurogenesis and remote spatial memory by neutralization of α2AP

**Fig. 1:**
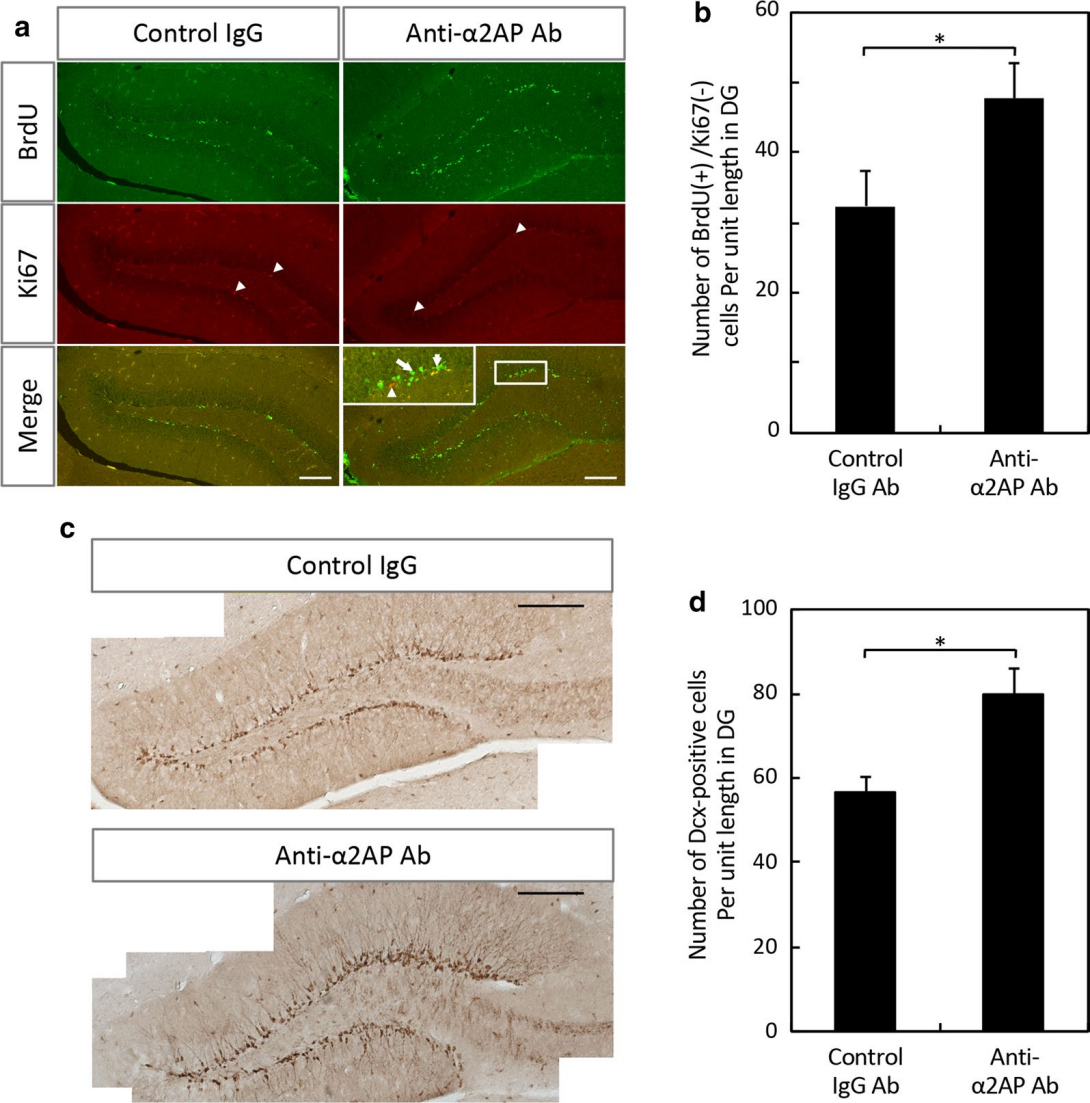
The effects of anti-α2AP neutralizing antibodies on adult hippocampal neurogenesis. **a** Representative images of immunostaining of BrdU and Ki67. Anti-α2AP neutralizing antibodies or control IgG were intracerebroventricularly injected in 12-week-old C57BL/6J mice 2 h after the first intraperitoneal injection of BrdU, and BrdU was administered at 24-h intervals for 7 days. Coronal brain slices were immunostained with antibodies. Arrow heads indicate Ki67^+^ cells, and arrows indicate examples of BrdU^+^/Ki67^−^ cells. Scale bar: 50 μm. **b** The numbers of BrdU^+^/Ki67^−^ cells in the DG were counted in a blinded manner. **c** Representative images of immunostaining of Dcx. Scale bar: 200 μm. **d** The numbers of Dcx^+^ cells in the DG were counted in a blinded manner. The values represent the means ± S.E. (control IgG: n = 4, α2AP Ab: n = 5). Statistical significance was evaluated using Student’s *t*-test. *P < 0.05

**Fig. 2:**
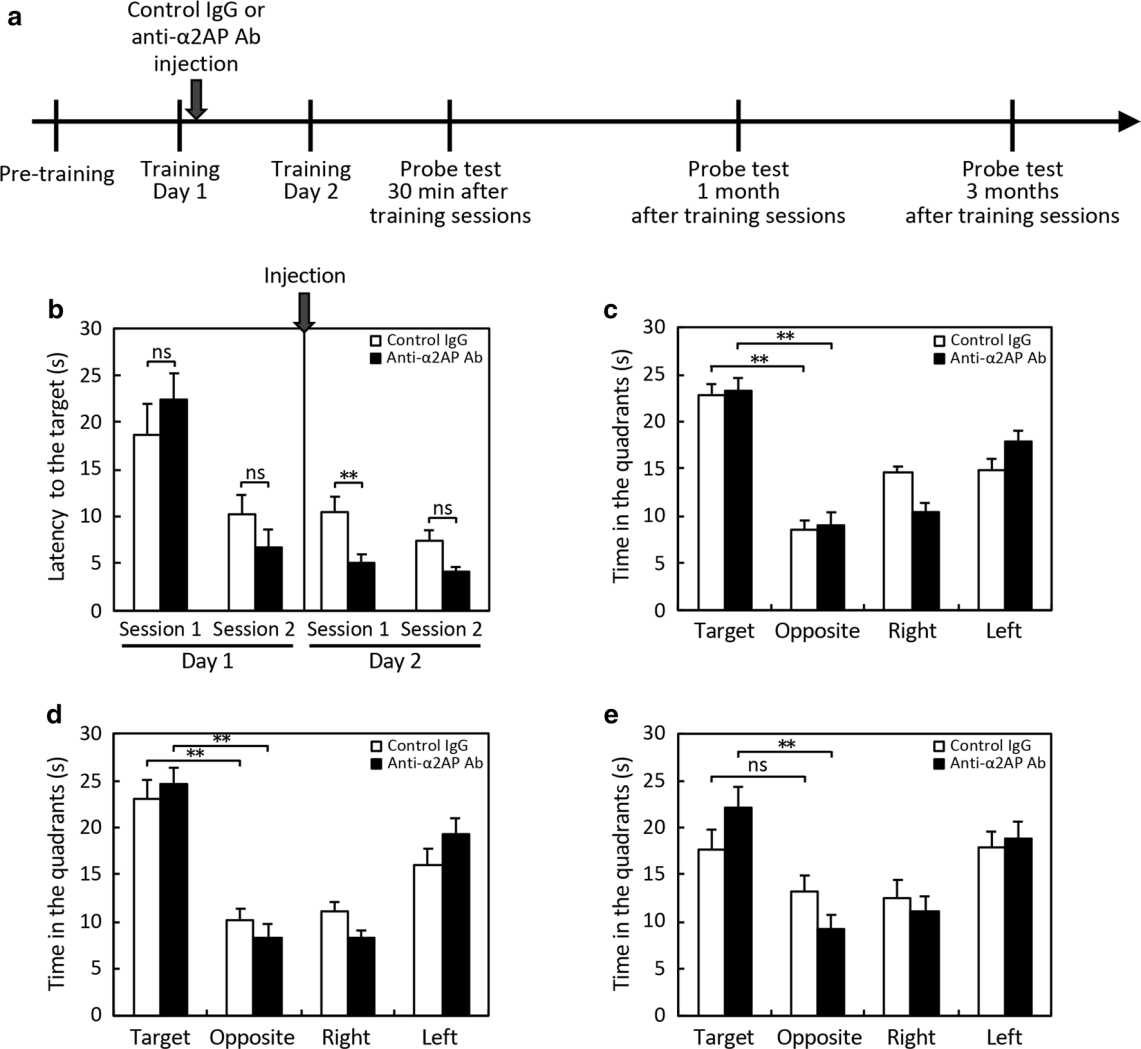
The Effects of anti-α2AP neutralizing antibodies on spatial memory. **a** Anti-α2AP neutralizing antibodies or control IgG were intracerebroventricularly injected in 12-week-old C57BL/6J mice after the first day of training in the MWM test. On the second day, mice were repeatedly trained, and then the probe tests were performed 30 min, 1 month and 3 months later. **b** The results of the training sessions. The latency to the target in each trial was measured. The values represent the mean values of 4 trials in each session. **c**–**e** The results of the probe tests at 30 min (**c**), 1 month (**d**) and 3 months (**e**) after training. The time in each quadrant was measured. The values represent the mean ± S.E. (control IgG: n = 10, α2AP Ab: n = 9). Statistical significance was evaluated using an ANOVA with an LSD post-hoc test. **P < 0.01, *ns* non-significant

To first determine whether α2AP mediates adult neurogenesis in the dentate gyrus (DG) of the hippocampus, we examined the effect of a neutralizing antibody against α2AP on neurogenesis. The number of BrdU-positive and Ki67-negative cells that had exited the cell cycle was significantly increased in the DG of the anti-α2AP antibody-injected mice in comparison to that of the control mice, although there were few Ki67-positive cells, proliferating cells, in the DG of the mature adult mice (Fig. 1a, b). We also showed that the number of Dcx-positive cells, which are immature neurons, was increased by neutralization of α2AP (Fig. 1c, d). As adult hippocampal neurogenesis is essential for long-term spatial memory [21, 26], we next examined whether α2AP was involved in the remote spatial memory in an MWM test. During the establishment of long-term memory, memory consolidation is a critical process by which newly acquired and labile short-term memory is transformed into a stable long-term memory [27]; and the reconsolidation after retrieval is required to maintain, strengthen, and update an acquired memory [28, 29]. Therefore, we injected the anti-α2AP antibody after the first day of training in the MWM test and evaluated the effect of neutralization of α2AP on the remote spatial memory (Fig. 2a). On the first day of training sessions, the latency to the platform in the two experimental groups (the anti-α2AP antibody-injected and control groups) did not differ to a statistically significant extent (Fig. 2b). After the first day of training, we intracerebroventricularly injected an anti-α2AP antibody or control IgG. On the second day, the latency to the platform in the first session in the anti-α2AP antibody-injected group was remarkably shorter in comparison to the control group (Fig. 2b). However, in the probe test at 30 min after training, the time in each quadrant did not differ between the anti-α2AP antibody-injected mice and control mice, and—in both groups of mice—the time in the target quadrant where the platform had been present was significantly longer in comparison to the other quadrants (Fig. 2c), indicating that both groups of mice remembered where the platform had been. The swimming velocity in the anti-α2AP antibody-injected mice and control mice were 17.1 ± 0.5 and 15.6 ± 0.6 cm/s, respectively, indicating that the swimming velocity was not affected by the injection of the anti-α2AP antibody. In the probe test at 1 month after the training sessions, the time in the target quadrant was significantly longer in comparison to the other quadrants in both groups of mice (Fig. 2d). However, in the probe test performed 3 months later, the time that the anti-α2AP antibody-injected mice spent in the target quadrant was still significantly longer than that in the opposite quadrant, while in the control mice, there was little difference in the time spent in the target quadrant and in the opposite quadrant (Fig. 2e). These results indicate that the neutralization of α2AP enhances adult hippocampal neurogenesis and spatial memory retention and recall.

### Reduction of hippocampal neurogenesis and remote spatial memory by excess α2AP

**Fig. 3:**
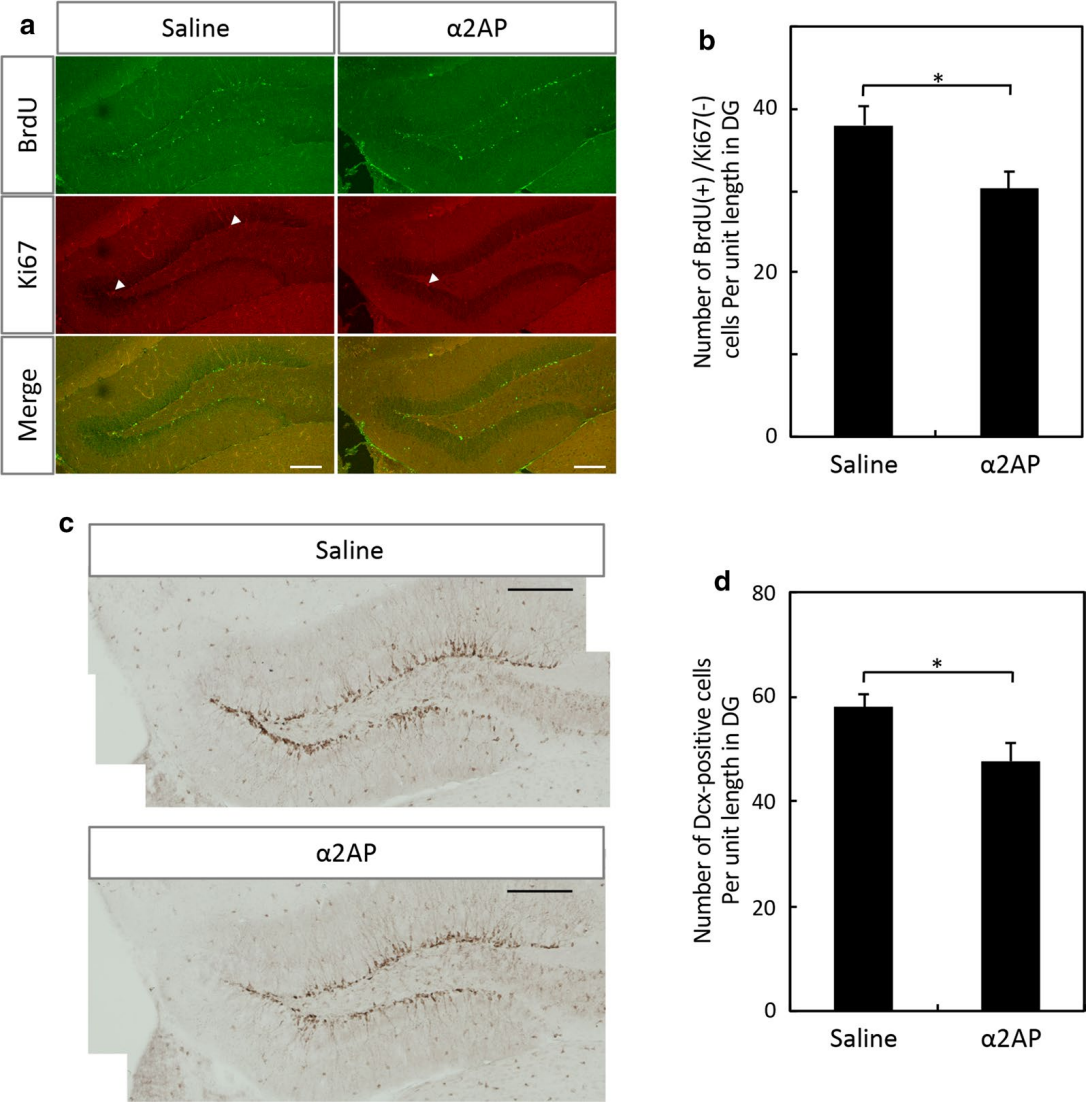
The effects of the injection of α2AP on adult hippocampal neurogenesis. **a** Representative images of immunostaining of BrdU and Ki67. α2AP or saline was intracerebroventricularly injected in 12-week-old C57BL/6J mice, 2 h after the first intraperitoneal injection of BrdU, and BrdU was administered at 24-h intervals for 7 days. Coronal brain slices were immunostained with antibodies. Arrow heads indicate Ki67^+^ cells. Scale bar: 50 μm. **b** The numbers of BrdU^+^/Ki67^−^ cells in the DG were counted in a blinded manner. **c** Representative images of immunostaining of Dcx. Scale bar: 200 μm. **d** The numbers of Dcx^+^ cells in the DG were counted in a blinded manner. The values represent the mean ± S.E. (saline: n = 5, α2AP: n = 6). Statistical significance was evaluated using Student’s *t*-test. *P < 0.05

**Fig. 4:**
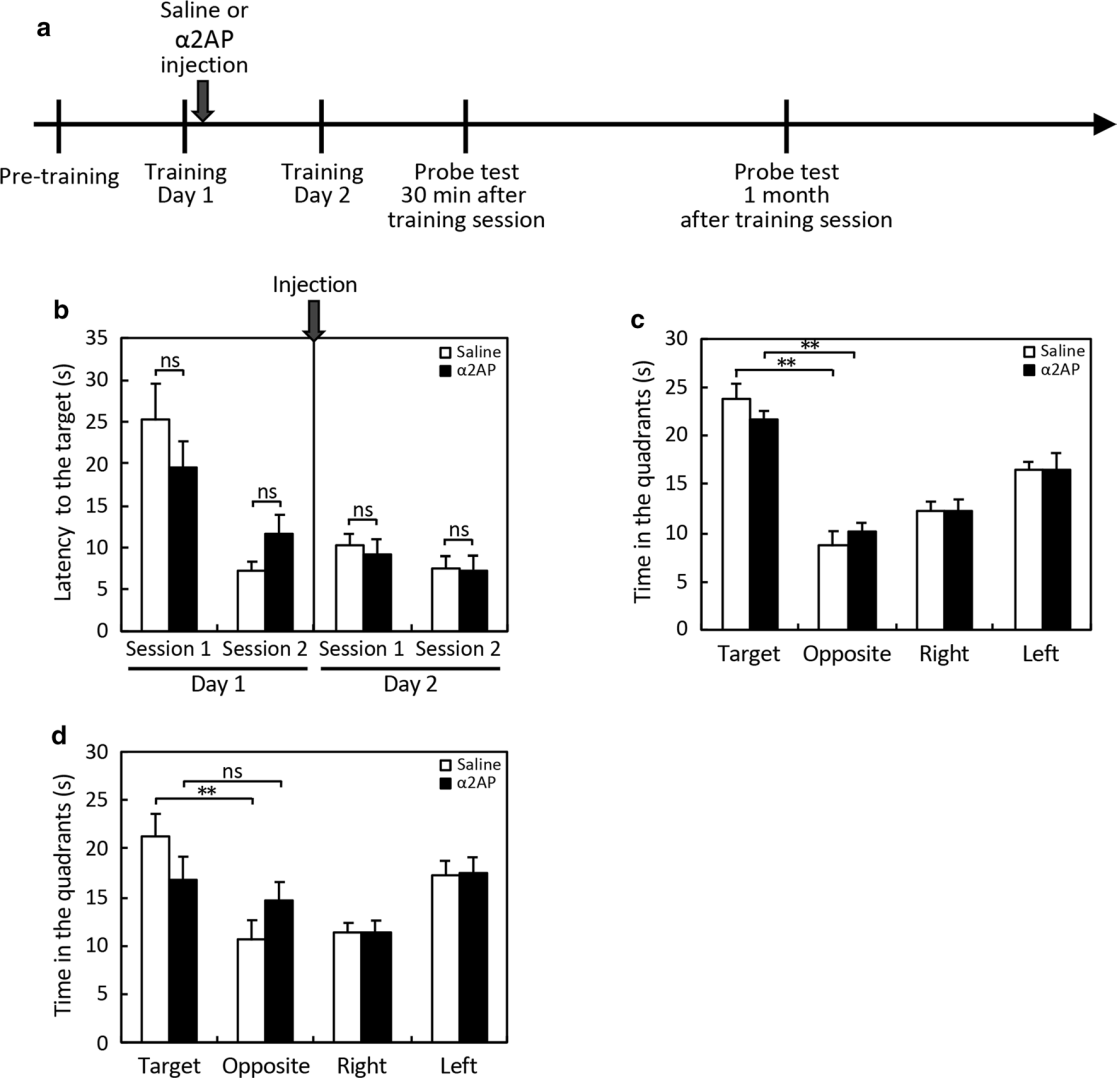
The effects of the injection of α2AP on spatial memory. **a** α2AP or saline was intracerebroventricularly injected in 12-week-old C57BL/6J mice after the first day of training in the MWM test. On the second day, mice were repeatedly trained, and then the probe tests were performed 30 min and 1 month later. **b** The results of the training sessions. The latency to the target in each trial was measured, and the values represent the mean values of 4 trials in each session. **c** and **d** The results of the probe tests performed 30 min (**c**) and 1 month (**d**) after training. The time in each quadrant was measured. The values represent the mean ± S.E. (saline: n = 8, α2AP: n = 10). Statistical significance was evaluated using an ANOVA with an LSD post-hoc test. **P < 0.01

We next investigated the effect of α2AP injection on adult hippocampal neurogenesis and the spatial memory process. The number of BrdU-positive and Ki67-negative cells in the DG was significantly decreased by the injection of α2AP in comparison to the control mice (Fig. 3a, b). Accordingly, the number of Dcx-positive cells was decreased by excess α2AP (Fig. 3c, d). α2AP was injected into the brain after the first day of training in the MWM test. Probe tests were performed on the second day of training (30 min after training), and then at 1 month (Fig. 4a). In the training for the MWM test, there was little difference in the latency to the platform between the α2AP-injected mice and the control mice (Fig. 4b). In the probe test at 30 min after training, the time in the target quadrant was longer than that in the other quadrants in both groups of mice, and the time in each quadrant did not differ between them, indicating that both mice remembered where the platform had been (Fig. 4c). The swimming velocity of the α2AP-injected mice and control mice did not differ to a statistically significant extent (16.5 ± 0.5 and 16.4 ± 0.7 cm/s, respectively). In the probe test at 1 month after the training sessions, there was no difference in the time that the α2AP-injected mice spent in each quadrant, while the time that the control mice spent in the target quadrant was markedly longer than that in the opposite quadrant (Fig. 4d). These results suggest that excess α2AP suppresses hippocampal neurogenesis, as well as spatial memory retention and recall.

### The amount of α2AP in the brain was negatively correlated with working spatial memory

**Fig. 5:**
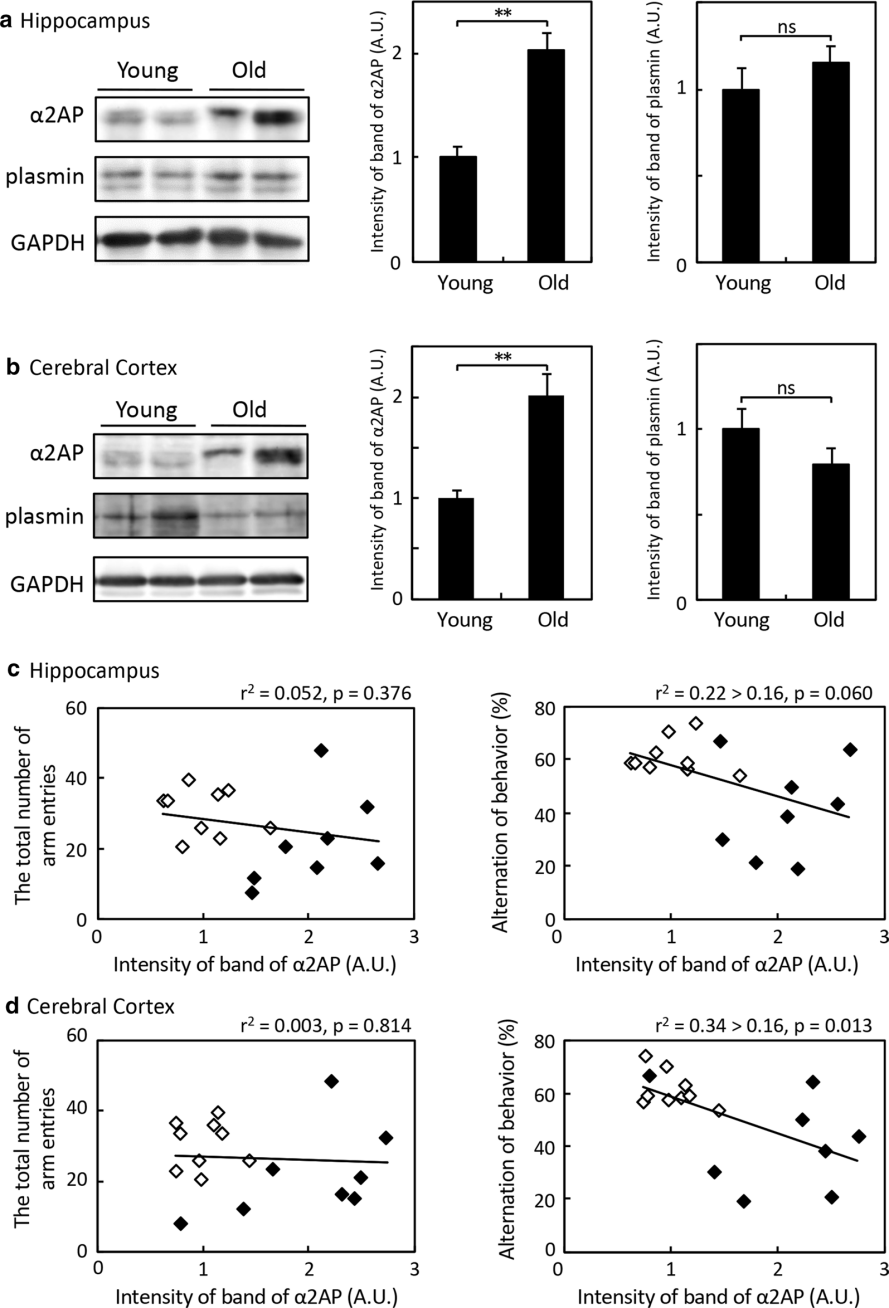
Correlation of α2AP levels in the brain and spatial working memory between young and old mice. **a** and **b** The levels of α2AP and plasmin relative to those of GAPDH in the hippocampus and cerebral cortex of young and old C57BL/6J mice were determined by Western blotting (young mice: 12–16 weeks of age, old mice: > 25 months of age). The band intensity was measured using the NIH ImageJ software program and normalized to that of GAPDH. The values were divided by the mean of the normalized intensity of young mice. The bar graphs represent the mean ± SE (arbitrary units: A.U., young mice: n = 8, old mice: n = 9). Statistical significance was evaluated using Student’s *t*-test. **P < 0.01. **c** and **d** The relative intensity of α2AP to GAPDH and the scores of the Y-maze test were analyzed by Pearson’s correlation test. Correlation was evaluated using contribution rate (r^2^): values of r^2^ > 0.16 were considered to indicate a correlation. The values of young mice and old mice were presented with a blank rhombus and filled rhombus, respectively

The degree of α2AP levels and functions in the brain was suggested to affect the hippocampus-dependent spatial memory. Thus, in order to elucidate the relationship between the α2AP levels and brain aging accompanied by cognitive decline, we first compared the levels of α2AP in the brain between young and old mice. The expression of α2AP in both the hippocampus and cerebral cortex in old mice was remarkably higher in comparison to young mice, while there was no significant difference in the expression of plasmin (Fig. 5a, b). The relative amount of α2AP in the cerebrospinal fluid in old mice tended to be higher than that in young mice (young mice: 1.0 ± 0.4, old mice: 1.5 ± 0.2; n = 4/group), while the α2AP level in the plasma in old mice was almost the same as that in young mice (young mice: 10.4 ± 1.8, old mice: 12.5 ± 0.9 μg/mL; n = 4/group).

We next analyzed correlations between the relative levels of α2AP in the hippocampus and cerebral cortex, and spontaneous activity and the working spatial memory scores in the Y-maze test (Fig. 5c, d). The total numbers of arm entries and the alteration of behavior, indicating spontaneous activity and working spatial memory score respectively, were significantly lower in old mice in comparison to young mice (Additional file 1: Fig. S1). There was no relationship between the total numbers of arm entries and the α2AP levels in the hippocampus or cerebral cortex (Fig. 5c, d). On the other hand, there was a negative correlation between the alteration of behavior and the α2AP levels, which was stronger in the cerebral cortex. These results suggest that an increase in α2AP in the hippocampus and cerebral cortex is likely to be associated with aging-dependent cognitive decline. We also analyzed the spatial working memory in old mice before and after the intracerebroventricular injection of an anti-α2AP neutralizing antibody and its control IgG. Unexpectedly, the spatial working memory was impaired after the injection of control IgG in old mice, but not in young mice, and this neurotoxic effect in old mice was suppressed by an anti-α2AP antibody (Additional file 2: Fig. S2). In contrast, young mice exhibited enhanced spatial working memory after the neutralization of α2AP. Although the neutralization of α2AP was potentially protective against brain injury in old mice, the effects of an anti-α2AP neutralizing antibody on age-related cognitive decline could not be elucidated.

### The reduction of aging-dependent oxidative stress and inflammation in the hippocampus by α2AP deficiency

**Fig. 6:**
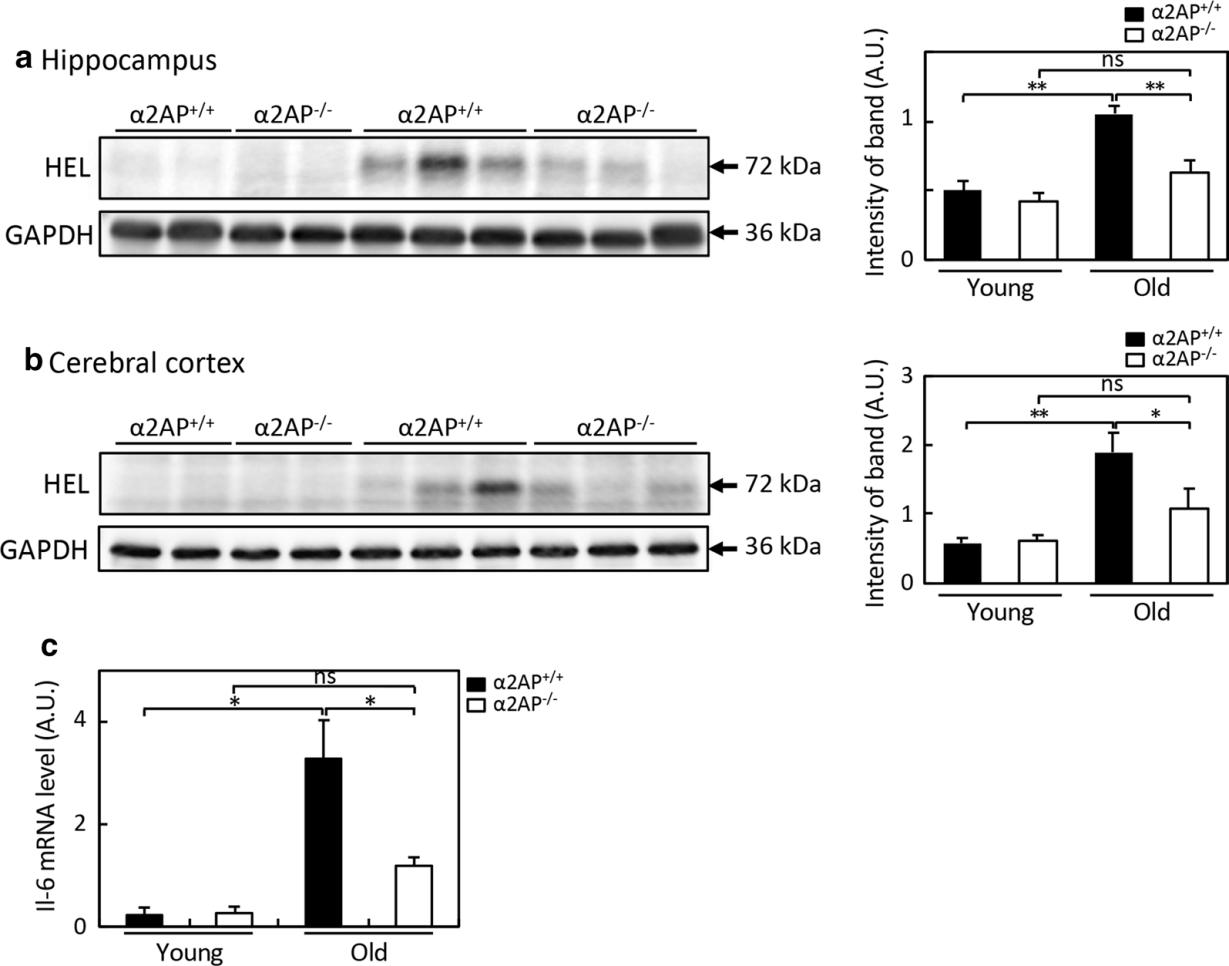
The effects of α2AP deficiency on age-dependent oxidative stress and neuroinflammation. **a** and **b** The levels of 13-HPODE-adducted protein relative to those of GAPDH in the hippocampus and cerebral cortex of young and old WT and α2AP^−/−^ mice were determined by Western blotting (young mice: 11 weeks of age, old mice: 60 weeks of age). The relative intensity was measured using the NIH ImageJ software program and normalized to that of GAPDH. The bar graphs represent the mean ± SE (young WT mice: n = 5, young α2AP^−/−^ mice: n = 4, old WT mice: n = 5, old α2AP^−/−^ mice: n = 4). **c** The levels of IL-6 mRNA in the hippocampus were shown (young mice: n = 7, old mice: n = 5). Statistical significance was evaluated by an ANOVA with an LSD post-hoc test. *P < 0.05., **P < 0.01

The theory of oxidative stress is widely considered to be a mechanism underlying age-related cognitive decline [23, 30]. In order to understand the role of α2AP in aging-dependent oxidative stress, we compared the degree of oxidative stress in the hippocampus and cerebral cortex of young and old WT and α2AP^−/−^ mice. The degree of oxidative stress was assessed by detecting the levels of 13-hydroperoxyoctadecanoic acid (13-HPODE)-modified proteins, which reacts specifically with an anti-HEL antibody (Fig. 6a, b). In the hippocampus and cerebral cortex of WT mice, the intensity of the 13-HPODE-modified protein was higher in old mice than in young mice. However, in α2AP^−/−^ mice, the age-related increase in the levels of the 13-HPODE-modified proteins was significantly reduced in the hippocampus and cerebral cortex. Thus, these results suggest that α2AP is involved in aging-dependent oxidative stress in the brain.

In addition, to examine whether α2AP was involved in the aging-dependent neuroinflammation, we performed real-time PCR analyses to detect proinflammatory cytokines. The expression of IL-6 mRNA in the hippocampus, but not in the cerebral cortex, was remarkably increased in old mice in comparison to young mice, while there was no significant difference in the expression levels of IL-1β or TNF-α mRNA in the hippocampus or the cerebral cortex between young and old mice (Additional file 3: Fig. S3). It is noteworthy that the age-related increase in the expression of IL-6 mRNA in the hippocampus was significantly inhibited in α2AP^−/−^ mice (Fig. 6c), indicating that α2AP mediates age-dependent hippocampal proinflammation.

## Discussion

While there are some inconsistencies in reports about the effects of manipulating neurogenesis on the hippocampus-dependent cognitive function, the majority of rodent studies suggest that adult hippocampal neurogenesis is involved in spatial memory, fear contextual memory, and pattern separation [31]. The ablation of hippocampal neurogenesis causes a deficit in remote spatial memory formation but not in recent memory formation, suggesting that neurogenesis is essential for long-term spatial memory [21, 26]. In the present study, we have demonstrated that neurogenesis in the DG was significantly enhanced by neutralization of α2AP (Fig. 1). Accordingly, anti-α2AP antibody-injected mice exhibit enhanced long-term retention in comparison to the control mice (Fig. 2). It is noteworthy that the neutralization of α2AP also enhanced short-term retention during training of the MWM test (Fig. 2). In contrast, excess α2AP reduced hippocampal neurogenesis, and caused the impairment of remote spatial memory formation but not recent memory formation (Figs. 3, 4). These results clearly suggest that α2AP is a negative regulator of hippocampal neurogenesis and remote spatial memory. Considering that memory retention was affected by the injection of α2AP or anti-α2AP antibody during training, α2AP probably modulate the memory consolidation and/or reconsolidation to form remote memory. The trisynaptic circuit in the hippocampus (layer II of entorhinal cortex → DG → CA3 → CA1) is crucial for memory consolidation to form remote memory, and newborn neurons within the DG form synaptic connections and are incorporated into the existing hippocampal circuits [32]. Thus, α2AP might modulate the numbers of newborn neurons integrated into the existing hippocampal circuits during memory formation.

Our previous study demonstrated that α2AP^−/−^ mice exhibit an impaired cognitive function, including spatial memory and fear conditioning memory, in comparison to WT mice [19], suggesting that α2AP is critical for the development of the cognitive function. We also showed that the dendritic growth in hippocampal neurons is impaired by the deletion of α2AP gene [18]. Given other previous reports describing the toxic effects of excess plasmin on the neuronal functions [9–11, 16, 17], in α2AP^−/−^ mice, failure in the regulation of plasmin activity in the brain may lead to the disruption of appropriate synaptic connections and neurotoxicity. Indeed, our supplementary experiment showed that a single injection of plasmin tended to cause impaired remote spatial memory (Additional file 4: Fig. S4). On the other hand, the present study has shown that transient inhibition of α2AP in the brain enhances remote spatial memory, and conversely, that excess α2AP reduces it (Figs. 2, 4). These results suggest that a transient increase in plasmin activity is necessary for remote spatial memory. Given that the production of mature BDNF by plasmin is essential for long-term hippocampal plasticity [12], increased plasmin activity through the inhibition of α2AP might promote the production of mature BDNF, leading to the enhancement of remote spatial memory, although other possible mechanisms by which α2AP regulates spatial memory, which do not involve the inhibition of plasmin, also needed to be elucidated.

We next aimed to understand the roles of α2AP in the cognitive decline caused by brain aging. We found a markedly higher amount of α2AP in the cerebral parenchyma of old mice in comparison to young mice and a negative correlation between the amount of α2AP and working spatial memory (Fig. 5). As it was additionally shown that excess α2AP impairs the cognitive function in young mice (Fig. 4), the increase in the levels of α2AP in the brain with age might cause the cognitive decline associated with aging. To determine whether α2AP is involved in aging-dependent cognitive decline, we analyzed the effects of the neutralization of α2AP on spatial working memory in old mice; the impairment of working memory was induced simply by the injection of the control IgG, while this working memory impairment was not found in young mice (Additional file 2: Fig. S2). The inhibition of α2AP seems to have protective effects against brain injury in old mice; however, in order to determine whether or not α2AP is involved in age-related cognitive decline, it is still necessary to find a way to inhibit α2AP without inducing brain damage in old mice; for example, a small molecule compound or a nanobody could be utilized.

The present study also demonstrated that α2AP contributes to aging-dependent oxidative stress and an increase in the expression of IL-6 mRNA in the hippocampus (Figs. 5, 6). Numerous studies have highlighted that associations between oxidative stress and brain damage and impaired neuronal plasticity leads to aging-dependent cognitive decline [23, 33]. In addition, IL-6 mediates the activation of neuronal NADPH oxidase and cognitive impairment in old mice [34]. Some clinical studies have reported that higher blood levels of IL-6 may be associated with lower white matter integrity, neural activity and cognitive functions in older people [35, 36]. These previous reports support that oxidative stress and an increase in the production of IL-6 are critical factors for brain aging. Furthermore, one study demonstrated that the chronic injection of α2AP into the medial prefrontal cortex inhibits the NGF maturation induced by plasmin, causing cholinergic degeneration and cognitive impairment [37]. Thus, an age-related increase in the amount of α2AP in the brain is suggested to induce brain oxidative stress and neuroinflammation, and to reduce the production of mature NGF—thereby possibly causing impaired neuronal plasticity, decreased hippocampal neurogenesis and neuronal loss, followed by cognitive decline.

A recent preliminary study reported that the plasma levels of α2AP are high in unhealthy octogenarians with cognitive decline, functional dependency and malnutrition in comparison to healthy octogenarians [38]. In contrast, the present study showed that there was little difference in the plasma α2AP levels of young and old mice, although the amount of α2AP in the brain of old mice was markedly increased in comparison to young mice. This inconsistency in the plasma levels of α2AP suggests a difference in the α2AP levels between normal brain aging and pathological brain aging. Hence, plasma α2AP could be a useful marker of the progression of aging-related cognitive disorders, including dementia.

In summary, we demonstrated that α2AP is a crucial negative regulator of adult hippocampal neurogenesis and remote spatial memory. Moreover, we elucidated that the amount of α2AP in the brain is negatively correlated with the cognitive function, and that α2AP is a critical factor of the brain oxidative stress and hippocampal proinflammation associated with aging. Considering these findings, together with our previous finding that α2AP^−/−^ mice exhibit impairments of the cognitive function and dendritic growth, the levels of α2AP in the brain are likely to fluctuate but gradually increase with age to affect the neuronal functions. Although further research is needed to elucidate the spatiotemporal expression of α2AP in the brain and its relationship with the neuronal functions, our present findings provide new insight into the physiological and pathological roles of α2AP in the brain, and furthermore, suggest that α2AP is a potential target for the effective regulation of healthy brain aging.

## Supplementary methods

Intracerebroventricular injection in mice performed the Y-maze test.

Mice were anesthetized with a combination anesthetic composed of 0.75 mg/kg of medetomidine, 4.0 mg/kg of midazolam and 5.0 mg/kg of butorphanol. A guide cannula (AG-4; Eicom) was implanted into the lateral right ventricle (0.2 mm caudally and 1.0 mm laterally to the bregma; and 1.9 mm vertically from the brain surface), fixed to the skull with dental cement, and then occluded with a dummy cannula (AD-4; Eicom). The mice were returned to their home cage and allowed to recover for 1 week. The Y-maze test was performed 1 week after the ventricular cannulation. On the next day of the Y-maze test, an injection cannula (AMI-4.5; Eicom) was connected through polyethylene tubing to a Hamilton syringe that had been preloaded with 0.1 μg/μL of an anti-α2AP neutralizing goat antibody (R&D System) or normal goat IgG control (R&D System), and inserted into the guide cannula in the awake mice. Each solution was injected in a total volume of 20 μL. On the next day, the Y-maze test was performed again.

## Supplementary information

**Additional file 1: Figure S1**. *Impaired spatial working memory in old mice in comparison to young mice*. The Y-maze test was performed in young and old C57BL/6J mice (young mice: 12-16 weeks of age, n = 8; old mice: >25 months of age, n=9). The mice were placed in the center and allowed to explore the apparatus for 8 min. The alteration of behavior was calculated as the ratio of the number of alterations to the total number of arm entries minus 2. The values represent the mean ± S.E. Statistical significance was evaluated using Student’s t-test. *P < 0.05.

**Additional file 2: Figure S2**. *The effects of anti-α2AP neutralizing antibodies on spatial working memory in young and old mice*. The Y-maze test was performed before and after an intraventricular injection of anti-α2AP neutralizing antibodies or control IgG in young and old C57BL/6J mice (young mice: 11 weeks of age, control IgG: n=8, α2AP Ab: n=9; old mice: 60 weeks of age, control IgG: n=9, α2AP Ab: n=8). The values represent the mean ± S.E. Statistical significance was evaluated using a paired t-test. *P < 0.05.

**Additional file 3: Figure S3**. *Comparison of the levels of inflammatory cytokines in the brain between young and old mice*. The mRNA levels of IL-6, IL-1β and TNF-α in the hippocampus (A) and the cerebral cortex (B) were determined by real-time PCR (young mice: 12-16 weeks of age, n = 8; old mice: >25 months of age, n = 9). Statistical significance was evaluated using Student’s t-test. *P < 0.05.

**Additional file 4: Figure S4**. *The effects of excess plasmin on spatial memory*. (A) Plasmin or saline was intracerebroventricularly injected in 12-week-old C57BL/6J mice after the first day of training in the MWM test. On the second day, mice were repeatedly trained, and probe tests were performed 30 minutes and 1 month later. (B) The results of the training sessions. The latency to the target in each trial was measured. The values represent the mean values of 4 trials in each session. There was no difference in latency to the platform between the plasmin-injected mice and the control mice. (C) The results of the probe tests 30 minutes after training. The time in the target quadrant was longer than the other quadrants in both groups of mice, and the time in each quadrant did not differ between the two groups. The swimming velocity of the plasmin-injected mice and the control mice did not differ to a statistically significant extent (15.6 ± 0.9 and 15.2 ± 0.5 cm/s, respectively). (D) The results of the probe tests at 1 month after training. The time spent by the plasmin-injected mice in the target quadrant was significantly shorter in comparison to the control mice, although the time in the target quadrant was still longer than the time in the opposite quadrant in both groups of mice. (E) The values represent the mean ± S.E. (saline: n=8, plasmin: n=9). Statistical significance was evaluated using an ANOVA with an LSD post-hoc test. *P < 0.05, **P < 0.01.

## Data Availability

All data generated or analyzed during this study are included in this published article and its supplementary information files.

## Abbreviations

ACSF: Artificial cerebrospinal fluid
α2AP: α2-Antiplasmin
BDNF: Brain-derived neurotrophic factor
BrdU: 5-Bromo-2′-deoxyuridine
Dcx: Doublecortin
DG: Dentate gyrus
GAPDH: Glyceraldehyde 3-phosphate dehydrogenase
HEL: Hexanoyl-lysine
IL-6: Interleukin-6
IL-1β: Interleukin-1β
LTP: Long-term potentiation
MWM: Morris water maze
NGF: Nerve growth factor
TNF-α: Tumor necrosis factor-α
tPA: Tissue plasminogen activator
WT: Wild-type
