# Supplementary figures and images for: α2-Antiplasmin as a potential regulator of the spatial memory process and age-related cognitive decline
Source: Mol Brain. 2020 Oct 15;13:140. doi: 10.1186/s13041-020-00677-3 (PMC7566027; doi:10.1186/s13041-020-00677-3)

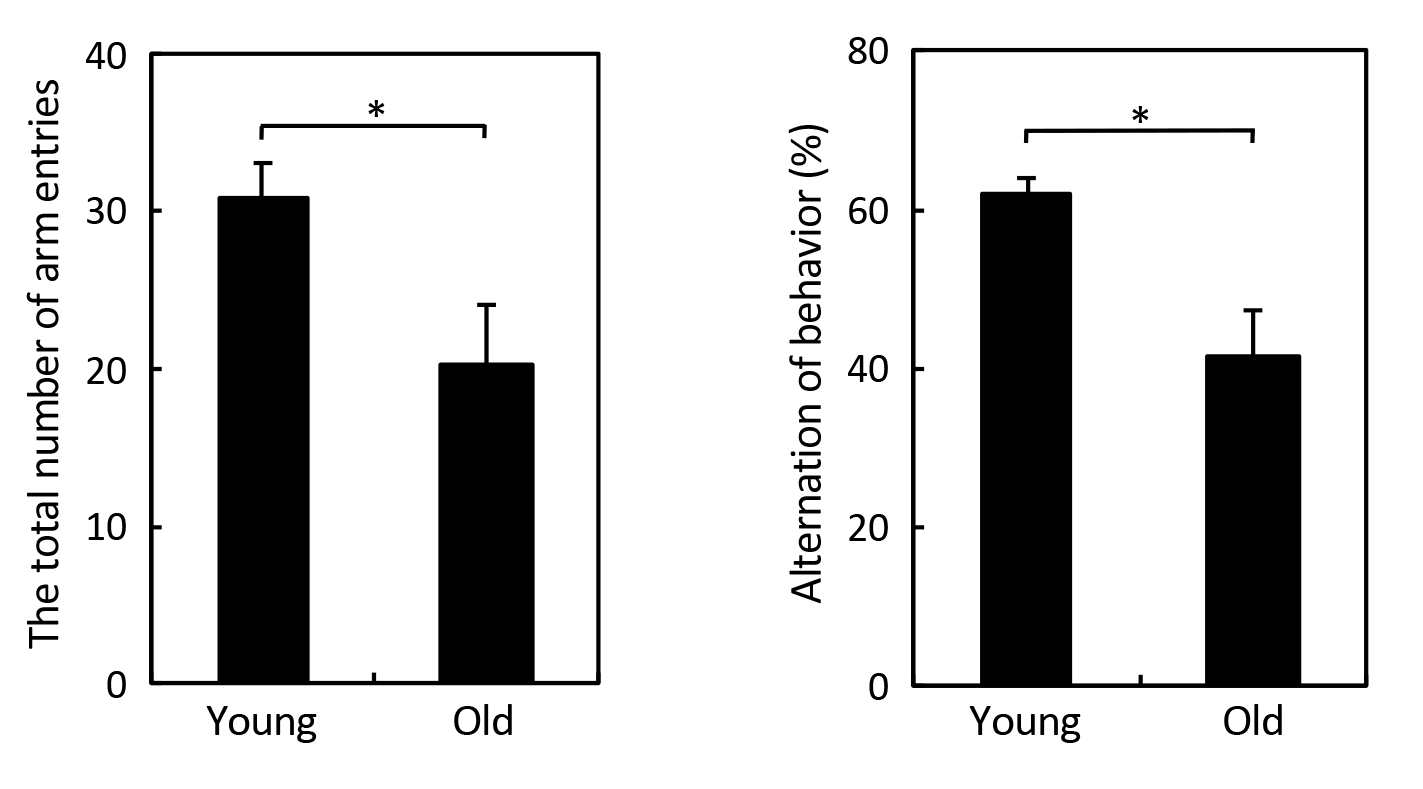

Supplement: Supplementary file 1 — Additional file 1: Figure S1. Impaired spatial working memory in old mice in comparison to young mice. The Y-maze test was performed in young and old C57BL/6J mice (young mice: 12-16 weeks of age, n = 8; old mice: >25 months of age, n=9). The mice were placed in the center and allowed to explore the apparatus for 8 min. The alteration of behavior was calculated as the ratio of the number of alterations to the total number of arm entries minus 2. The values represent the mean ± S.E. Statistical significance was evaluated using Student’s t-test. *P < 0.05. [file 13041_2020_677_MOESM1_ESM.tif]

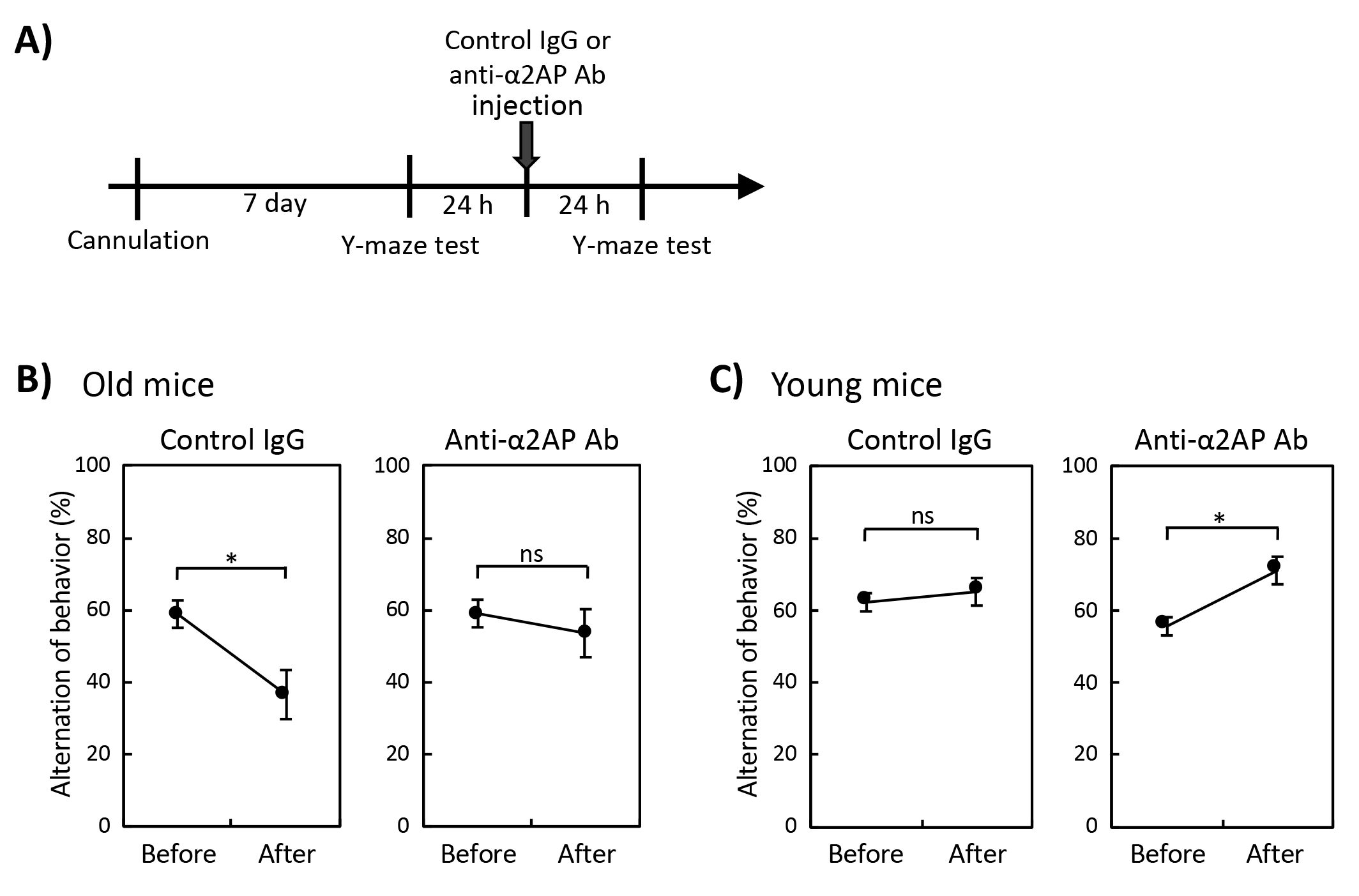

Supplement: Supplementary file 2 — Additional file 2: Figure S2. The effects of anti-α2AP neutralizing antibodies on spatial working memory in young and old mice. The Y-maze test was performed before and after an intraventricular injection of anti-α2AP neutralizing antibodies or control IgG in young and old C57BL/6J mice (young mice: 11 weeks of age, control IgG: n=8, α2AP Ab: n=9; old mice: 60 weeks of age, control IgG: n=9, α2AP Ab: n=8). The values represent the mean ± S.E. Statistical significance was evaluated using a paired t-test. *P < 0.05. [file 13041_2020_677_MOESM2_ESM.tif]

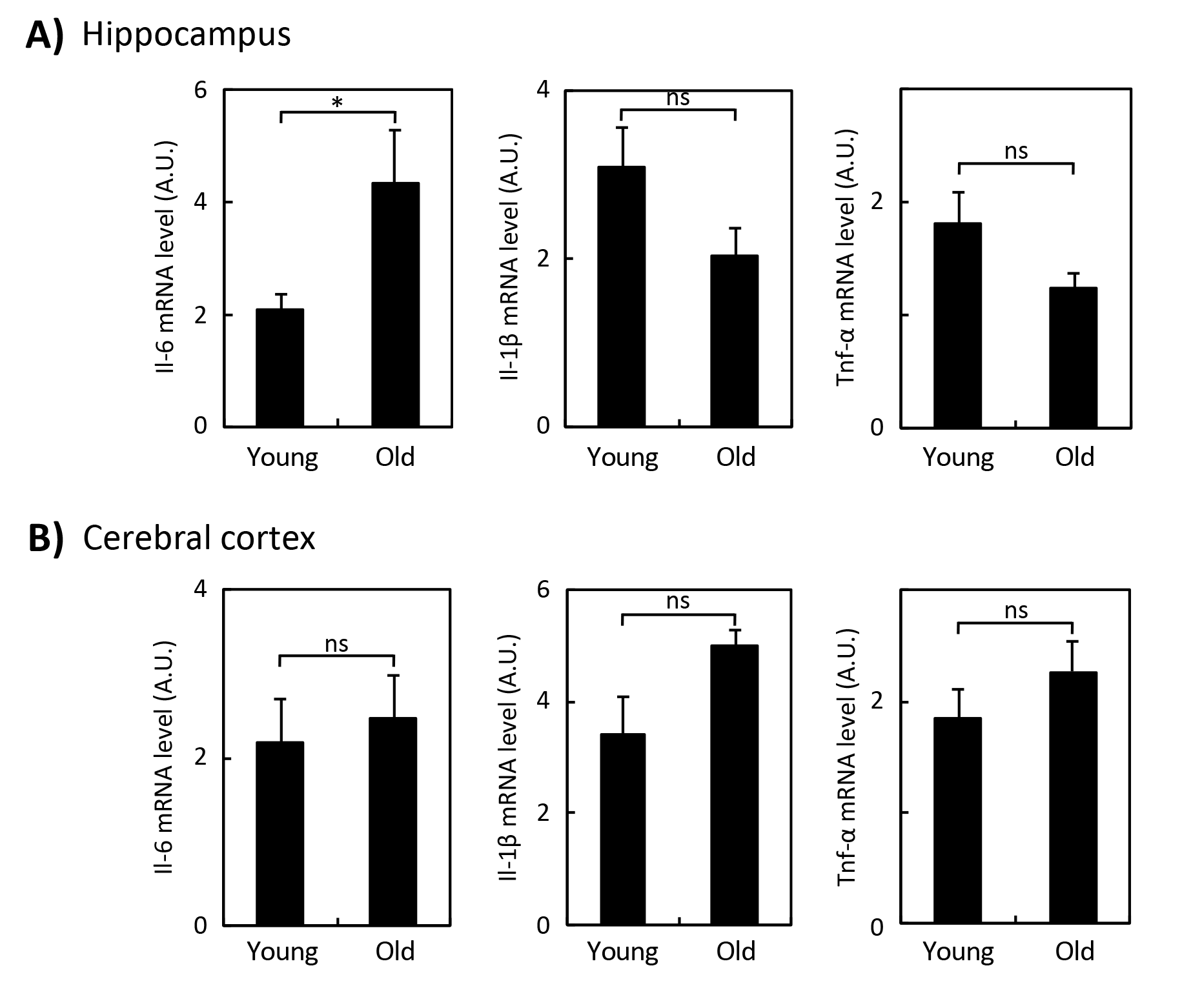

Supplement: Supplementary file 3 — Additional file 3: Figure S3. Comparison of the levels of inflammatory cytokines in the brain between young and old mice. The mRNA levels of IL-6, IL-1β and TNF-α in the hippocampus (A) and the cerebral cortex (B) were determined by real-time PCR (young mice: 12-16 weeks of age, n = 8; old mice: >25 months of age, n = 9). Statistical significance was evaluated using Student’s t-test. *P < 0.05. [file 13041_2020_677_MOESM3_ESM.tif]

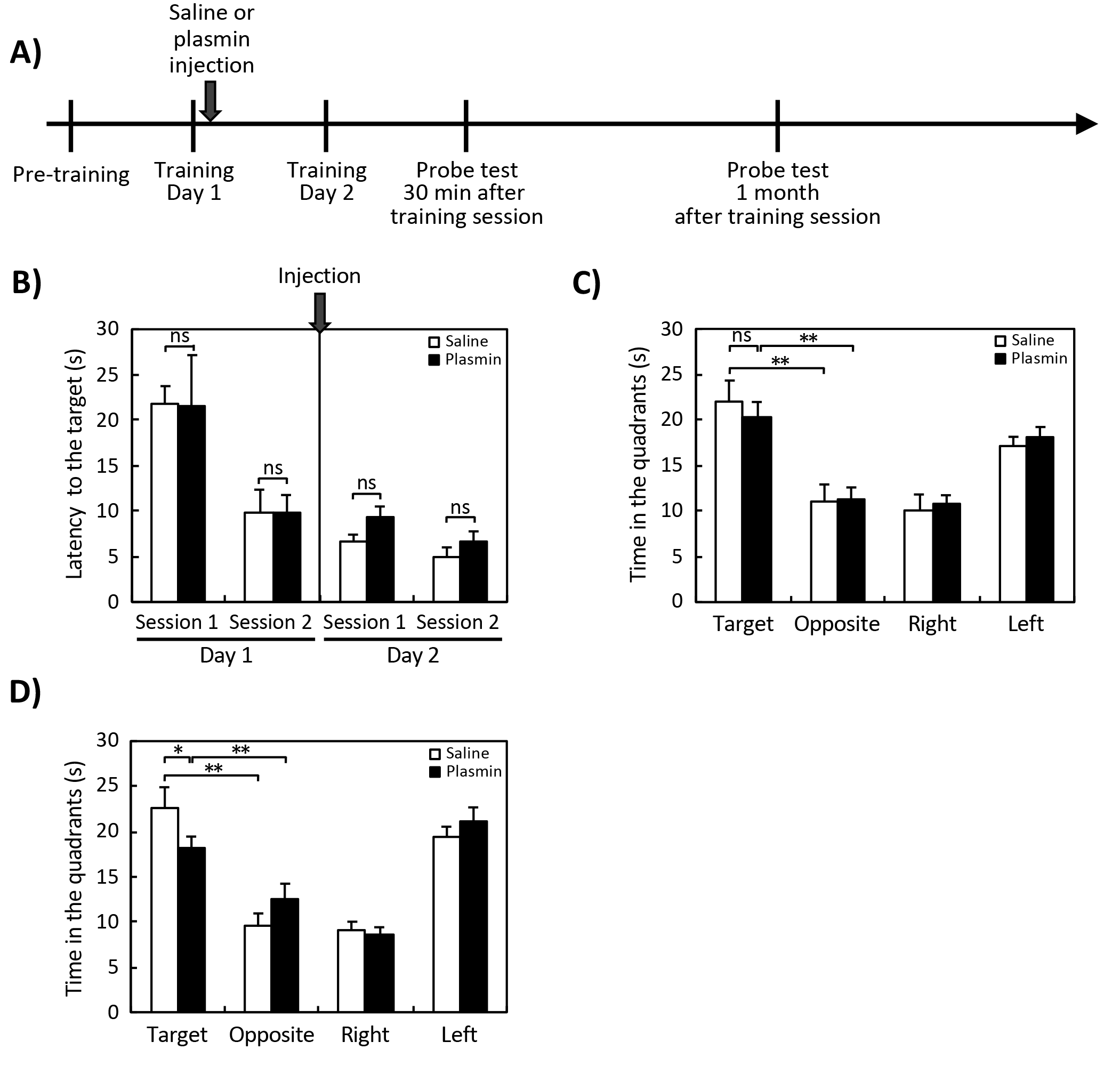

Supplement: Supplementary file 4 — Additional file 4: Figure S4. The effects of excess plasmin on spatial memory. (A) Plasmin or saline was intracerebroventricularly injected in 12-week-old C57BL/6J mice after the first day of training in the MWM test. On the second day, mice were repeatedly trained, and probe tests were performed 30 minutes and 1 month later. (B) The results of the training sessions. The latency to the target in each trial was measured. The values represent the mean values of 4 trials in each session. There was no difference in latency to the platform between the plasmin-injected mice and the control mice. (C) The results of the probe tests 30 minutes after training. The time in the target quadrant was longer than the other quadrants in both groups of mice, and the time in each quadrant did not differ between the two groups. The swimming velocity of the plasmin-injected mice and the control mice did not differ to a statistically significant extent (15.6 ± 0.9 and 15.2 ± 0.5 cm/s, respectively). (D) The results of the probe tests at 1 month after training. The time spent by the plasmin-injected mice in the target quadrant was significantly shorter in comparison to the control mice, although the time in the target quadrant was still longer than the time in the opposite quadrant in both groups of mice. (E) The values represent the mean ± S.E. (saline: n=8, plasmin: n=9). Statistical significance was evaluated using an ANOVA with an LSD post-hoc test. *P < 0.05, **P < 0.01. [file 13041_2020_677_MOESM4_ESM.tif]
